# ‘Deliver This Horse from Evil’: The Ritual Aspects of Responses to Veterinary Disease in the Late Middle Ages

**DOI:** 10.1093/shm/hkab042

**Published:** 2021-10-06

**Authors:** Sunny Harrison

**Keywords:** ritual, medicine, disease, veterinary, horse

## Abstract

While the importance of religious and magical healing practices in the Late Middle Ages is well established, the ritual aspects of veterinary medicine have so far not been thoroughly explored. This article addresses this lacuna through analysis of a corpus of charms, prayers, and other rituals that were used to cure a group of devastating contagious diseases that afflicted horses: animals that were often afforded complex, professional medical care in this period. It considers the semantic aspects and common features of this group of disease rituals alongside discussions of contagious illness in veterinary treatises, identifying a distinctive set of healing rituals and explaining why they were such a common response to enzootic disease. It argues that magical and religious healing were significant elements of medieval horse-care and that veterinary medicine has been overlooked as one of the key manifestations of ritual healing.

In their 2019 contribution to the *Routledge History of Medieval Magic*, Peter Murray Jones and Lea T. Olsan noted that the ‘ritual aspects of veterinary medicine’ present a significant gap in our understanding of magical healing. They argued that animal care provides a so far unclaimed opportunity to analyse a specific strand of healing magic; to identify the ‘incantations or rituals’ used and place them within the spectrum of remedies on offer for that condition.^1^ This lacuna in the study of medieval healing practices was created in part because historians of veterinary medicine have so far been unusually hostile to magical remedies and other ‘superstitious’ animal-healing practices. Scholars of medieval human medicine have largely accepted that creating rigidly separate categories of rational (i.e. scientific, university-educated), magical (charms), and religious (miracle-seeking and prayers) healing is erroneous and ignores the complex and ambiguous relationships between curative strategies. For the most part, charms and prayers are no longer seen as part of a ‘forceful opposition between paganism and Christianity’.^2^ Incantations and rituals, such as the Veronica charm in John of Gaddesden’s *Rosa Anglica*, were common parts of even a university-educated physician’s arsenal, and often deemed licit by church authorities.^3^ Although the specifics of magic’s rationality in the minds of its practitioners are still somewhat contested, magic, religion, and medicine are now viewed not as incompatible systems but as ‘different authorities’ with the same end goals.^4^

Veterinary historians on the other hand have often presented magical and religious remedies as broadly unscientific and inherently marginal.^5^ Charms and prayers are explained away as methods of last resort or peculiarities.^6^ Where these elements exist in horse medicine texts—which they do in abundance—they have been ignored or disregarded.^7^ Despite their prevalence, horse-healing rituals have been dismissed as superstitious aberrations and an irrational ‘part of the growing use of horses by poorer classes’.^8^ Treatises free of the ‘stains’ of sorcery have been emphasised as pure and ‘scientific’, even ‘rigidly experimental’ when compared with texts which are sullied by magical content.^9^ In 2013, Matthew Milner provided an exception to this trend in his discussion of a fifteenth-century charm for ‘founder’—a painful hoof inflammation. Milner argued that charms were not only rational responses to horse diseases, but also part of a ‘vernacular physics’ that situated ritualistic remedies between natural philosophy and medicine.^10^ Since 2013 there have been no further efforts to capitalise on Milner’s approach and analyse ritual healing as a fundamental, rational element of medieval veterinary medicine.

This article will begin to fill the gap in our understanding of medieval healing magic identified by Jones and Olsan by analysing a tradition of charms and performative rituals that were used to cure a set of highly contagious and potentially life-threatening horse diseases that were known variously in the Middle Ages as farcy or ‘worms’.^11^ These cures were a common feature of the horse medicine—or *hippiatric—*treatises that proliferated between c. 1250–1450.^12^ Analysing these remedies will help us to understand the place of ritual in the care of elite, managed equines, particularly by the class of horse-care practitioners known as ‘marshals’ that developed alongside the *hippiatric* tradition.^13^ This article disputes the argument that horse medicine was less inclined towards the use of magical and religious healing than human medicine—and was therefore a less superstitious and more rational practice—by demonstrating that charms, prayers, and other ritual cures were preserved in high-status treatises and used by a wide variety of horse-care practitioners without restriction to a particular group or status of user. By considering the features, modalities, and performative aspects of these remedies, we can see how the construction of these rituals was influenced both by their popular nosology—the categorisation or characterisation of the diseases—and by healing practices rooted in natural philosophy, magic, and miracle-seeking. This helps to explain why these rituals were so widely used as a response to farcy. In turn, this allows us to explore attitudes towards this common and yet so far largely unaddressed disease, thinking in particular about what motivated the use of ritual remedies and the potential impact of this illness on horses and their carers.

## Horse-Care, Animal Disease and Contagion

Late-medieval society relied heavily on horses; as the dominant agricultural and haulage animal they helped to increase productivity and decrease travel time.^14^ They were vital to transport, commerce and of course warfare.^15^ Elite horses were an integral part of the apparatus of the knightly aristocracy, as social vehicles but also as a necessary part of a largely itinerant lifestyle.^16^ The small markets in elite horses were linked to both international trade and noble gift exchange; and the breeding, training and development of elite horses was a skilled and labour-intensive process.^17^ All of this meant that replacing a horse was not a trivial matter. When a horse became sick its carers had a number of available options, if they were wealthy the horse might be placed under the supervision of occupational horse carers (stable masters and grooms). If they were of middling status, they would likely have access to marshals, who were taught through apprenticeship like barbers and other craftsmen and who disseminated their knowledge orally and through remedy collections.^18^ The horse’s carer might restrict its diet, administer a purgative or blood-letting, or give it a pharmaceutical preparation made from herbs, minerals and animal products. They might also use ritualistic medicines—charms, prayers and textual amulets—to alleviate symptoms and combat disease.

A group of ailments commonly known as farcy, worms or strangles were particularly likely to attract a ritual response.^19^ These were pernicious diseases that were often fatal, spread rapidly amongst herds, and according to the *hippiatric* writers could even be passed on to humans.^20^ Most animal diseases in the Middle Ages have not received the broad interdisciplinary attention of human illnesses such as leprosy or plague.^21^ Nor have medieval historians addressed the responses of animal-care practitioners to disease outbreaks in the same way as historians of later periods.^22^ Research into medieval animal diseases has so far focussed on sheep and cattle rather than elite animals such as horses, which were more likely to receive professional veterinary care.^23^ These studies have focused on the economic response to outbreaks, rather than magical or even medical reactions.^24^ They have concentrated on the kind of large-scale epizootics visible in chronicles and manorial accounts rather than persistent, enzootic disease that—whilst having less economic impact—were part of the quotidian duties of animal carers and are therefore quite visible in veterinary texts.^25^

Farcy was one of a number of related diseases including ‘worms’ or *vermis*, *vermis volatilis* and *vermis maior*, which *hippiatric* writers blamed on a swelling in the chest or groin which would then develop into one or other differential variety. These were all typified by pustules or eruptions across various parts of the body, which were compared to the fissures caused by an earthworm or an overstuffed sausage. Horse-doctors blamed farcy on excessive moistening of the flesh and increased production of fluids within the body, which erupted into the disease’s archetypal swellings and fissures. Jordanus Ruffus (d. 1256)—arguably the most important horse medicine writer in late-medieval western Europe—explained that *vermis* began in the chest or groin and then descended through the hips or shoulders until the entire legs became perforated with densely packed ulcers. A horse afflicted with the early stages of *vermis* could also develop other sometimes more serious diseases. If *vermis* spread to the horse’s head, it could lead to *vermis volatilis*, which caused frigid, oily mucous to pour out through the nostrils.^26^ If *vermis* never spread out from the chest, then it would instead become swollen and form a lethal tumour called *anticore* for its closeness to the heart.^27^ Another form of this disease called *guttam* presented either in the front of the horse or throughout the whole body, in which case it could only be cured through blood-letting.^28^

These diseases were usually explained through blockages around key glands in the chest and groin, which were caused by blood or fluids which were misdirected, putrified or overheated. This caused inflammation of the chest and limbs and the formation of ulcers, which erupted from the horse’s skin drawing out vital fluids. Jordanus Ruffus blamed *vermis* on ‘excessive hot humours’ collecting around the glands in a horse’s chest or groin. These then flowed into the legs, causing the characteristic ulcers to emerge.^29^ Laurentius Rusius (fl. c. 1340) believed that farcy was caused by putrefied blood being distributed around the body through the veins.^30^ Most of the *hippiatric* writers described a physiological system that can only partially be aligned with humoural theory—in which sickness and health are driven by the balance of four vital humours in the body: blood, phlegm, yellow bile or choler, and black bile. Jordanus explained illnesses such as *vermis* through blockages, inflammations and redirected ‘humores’ or fluids, but in a more mechanical sense than contemporary humoural medicine.^31^ Some later *hippiatric* writers developed Ruffus’s approach to disease aetiology to better reflect humoural theory. For instance, Magister Maurus (c. 1256–1300) surmised that *guttam* was caused by excess blood in the body, which becomes corrupted forming a ‘withered fleshy nodule’ in the chest. This nodule corrupts any fluids that flow through it, which become poisonous and are transformed into black bile that sinks due to its weightiness into the horse’s lower limbs, causing the legs to swell. There the black bile forms yellow, choleric ulcerations—the weeping, putrid sores that were characteristic of most types of farcy.^32^ Although these diseases were internal in nature and thought to be related to inflammation or misdirection of fluids, they required an external cause as well. This could be an accident or poor management, when a horse was ridden hard and then not cooled down properly or not bled regularly enough.^33^

The most pressing concern for horse carers faced with farcy was that the disease was contagious and easily transmissible from infected to uninfected horses through bites, smells and physical contact. The naturalist Albertus Magnus (d. 1280) believed that farcy was sometimes caused by an afflicted horse biting another, whereas Laurentius Rusius was more adamant that it was ‘usually caused by a horse consorting with other horses that have farcy’.^34^ Boniface of Calabria (fl. c. 1270) considered farcy to be ‘very contagious’; he listed it amongst other infectious diseases including strangles and cautioned against allowing horses with scabies to rub against other uninfected horses.^35^ Both the anonymous *Practica equorum* and Albertus Magnus warned that horses with strangles should be separated from other horses for fear of infection, but cautioned that even physically separated horses could transmit the disease over a shared fence through their wheezing breath.^36^ The fifteenth-century English *Boke of Marchalsi* noted that the ‘little farcy’ was caused by smelling a nearby horse that is infected, or by the wind from an infected horse. All of this had ramifications for the management of sick horses within a herd as horse carers were implored not to allow a sick horse to wander and infect other animals.^37^ If restricting physical contact between horses was insufficient because contagion was possible at longer distances via smells or the air, then sick horses would have to be isolated to avoid further infection.

In the case of a particularly serious outbreak horses might need to be placed into long-term isolation or restricted from entering towns and communities. In spring and summer 1298, the stable masters of Robert II of Artois at Cercamps-lès-Frévent sent four horses suffering from ‘le mal saint Eloy’ into isolation for several months, and another two in October.^38^ The relatively extreme precaution was provoked by fear of the disastrous potential of a disease spreading throughout the Count’s stables. We see similar concerns in other communities, such as in a 1497 miracle attributed to St. Magnus (d. 772) when a farmer was barred from entering the monastery at Füssen with a horse that was afflicted with *phagadena*, a disease that causes flesh-eating ulcers to appear on the skin. He finally managed to convince the monks to show mercy, but rather than allowing him to bring the horse to the altar they instead brought out ‘hay that had previously touched the relics of St. Magnus and whey that had been purified through prayer’.^39^ The monks’ unwillingness to allow the sick horse to enter the church reflects their fear of infectious diseases as well as a general anxiety around contagion.

In addition to being highly infectious, the prognosis for these illnesses was usually quite grave. Boniface of Calabria noted that a horse diagnosed with *vermis* would not recover without treatment.^40^ Jordanus Ruffus warned that a horse with *vermis volatilis* was unlikely to survive and that *anticore* was particularly lethal because it could overwhelm the heart.^41^ A surviving horse would likely be left scarred and therefore less valuable to an elite culture that viewed horses as part of their own self-expression.^42^ These diseases were considered severe enough to require professional aid. In 1380, the stables at Durham Priory paid a man 13 pence to cure a horse of farcy, and in 1284, Henry Marshal was paid 14 pence to treat a black warhorse belonging to Edward I using cautery and other curatives.^43^ In 1327, two of Edward III’s foals died of farcy, whilst under the care of William Marshal at Reading and in 1331 Arnald de Garcy (one of Edward’s stablemasters) paid 7 shillings and 6 pence for ‘diverse medicines’ for a horse with strangles.^44^ In 1396, during a farcy outbreak at the stable of Philip the Bold, Duke of Burgundy at Villiers, the Duke’s marshal Jehan de Pons was paid 30 francs to spend 87 days caring for the horses.^45^ Horse-doctors were also paid for magical remedies, for instance in 1420 when the stable of the French dauphin and regent at Pontereau de Mehun paid an unnamed horse carer six shillings and five pence to charm a Rouen carthorse that was suffering from farcy.^46^ These diseases were a persistent problem in the late Middle Ages; they could incapacitate, disfigure or kill animals as well as disrupting breeding and trading. Horse owners and horse carers responded with a variety of curative methods, notably a wide array of charms and other ritual remedies.

## Ritual Healing and Horse Medicine Treatises

Both horse carers and human healers used charms, prayers and other rituals in response to a broad array of illnesses. Rituals were offered in response to acute, episodic and chronic conditions including bleeding, lameness and fevers. They were used to counter behavioural dysfunctions that would render a riding or warhorse unusable and as a defence against magical control exerted by other humans or malevolent forces.^47^ However, the most common use of rituals in horse medicine was against the group of infectious diseases that included farcy. At their simplest, charms were ‘words or characters to be spoken or inscribed on objects’; within this, we find a wide variety of rituals from a simple *pater noster* to multi-stage procedures involving several charms, tools and symbols.^48^ Other remedies that do not fit this schema might nevertheless be deemed ‘performative rituals’, practices whose value stems in part from being eminently repeatable such that they acquire a ‘traditionality’ which brings authority and the power to heal or affect other meaningful change.^49^ The relationships between these remedies make it clear that the boundaries of categorisations such as magic, religion and science were often complicated and ambiguous.

Is it then sensible to mark out some remedies as magical or religious in nature and others as mundane? Consider two similar remedies for fistula in horses, from thirteenth-century northern Italy. The first instructs the healer to feed the horse pulverised viper mixed with quick lime and strong wine; the second calls for the horse to be fed a green meadow-frog that was collected whilst saying three *pater nosters*, dried out in the sun and ground into powder.^50^ They are both repeatable instructions involving ingesting ground herpetiles and are distinguished largely by the second remedy’s use of the *pater noster.* We could argue that the first remedy was a form of bizarre but nevertheless mundane pharmacy, whilst the latter remedy was clearly ritualistic, but to do so ignores the slippages between these categories.^51^ The doctrine of occult properties means that the pulverised viper and green-meadow frog could both be imbued with hidden powers that were within nature, but outside of natural comprehension.^52^ When a healer was instructed to recite a prayer to one or more holy figures, we might comfortably think of this as within the bounds of religion, but more complicated rituals challenge the distinction between magical and religious healing. A procedure against farcy contemporary to the remedies above instructed the healer to collect a golden oriole (a type of song-bird) whilst saying the *pater noster*, replacing the ‘us’ in ‘deliver us from evil’ with the name of the horse they wish to be healed.^53^ The *pater noster* was deemed a licit form of collection magic (an incantation chanted whilst collecting herbs or medicaments) by some church figures, but when it is used to collect a bird which is then tied around a horse’s neck was this an orthodox form of religious healing or curative magic?^54^ Was there always a clear distinction in the mind of the practitioner between the two, or were they just ‘different authorities’?^55^

The rituals discussed below are drawn from a selection of veterinary texts and medical compendia dating from the last quarter of the thirteenth to the end of the fifteenth centuries and originating mainly in Italy, France and England. This period saw the development of a tradition of horse-care treatises centred on *De medicina equorum* by Jordanus Ruffus. This text was one of the first new horse medicine treatises to be composed in Latin since Vegetius’ *Digesta artis mulomedicinae* (late fourth century), it was translated into at least nine languages, disseminated widely, and formed the basis for a tradition of *hippiatric* medicine that lasted until the development of veterinary colleges in the eighteenth century.^56^  *De medicina equorum* did not include charms or ritual healing, nor did several principal thirteenth and fourteenth-century *hippiatric* texts, notably those of Teodorico Borgognoni (d. 1295/6) and Laurentius Rusius. These authors did not state any clear disdain or dislike for magical material or criticise those that used it, magic and prayer are simply absent. Other contemporary horse medicine writers such as Jacopo Doro (c. 1275), Ubertus de Curtenova (c. 1250–1300) and Boniface of Calabria did include charms and rituals in their treatises so it is difficult to clearly identify a *hippiatric* bias against magic.^57^ When we examine the wider *hippiatric* tradition, it becomes clear that neither was lack of magic a generic convention of earlier *hippiatric* treatises, akin to the distinction between medical compendia and *practica*.^58^ Of the three examples cited above, Jacopo and Ubertus’ treatises might be thought of as remedy collections, though Ubertus’ proemium suggests a similar deontological approach to Jordanus. Boniface of Calabria’s text is clearly a *hippiatric* treatise, recognisably similar in form and theory to *De medicina equorum* and as the focus of several extremely valuable manuscripts it would be unwise to think of it as an outlier.^59^

Ritual remedies were often part of the process of compilation and editing that typified the *hippiatric* corpus following Jordanus Ruffus.^60^ Rituals were integrated into late-medieval veterinary manuals as marginal annotations, new scribal additions to older texts, and as fundamental parts of newer treatises and collections.^61^ One of the earliest thirteenth-century Latin witnesses of *De medicina equorum* employed a *pater noster* as a collection charm.^62^ The users of *hippiatric* texts were clearly interested in magical materials as they often annotated their treatises with charms as well as divinations such as the Sphere of Pythagoras.^63^ Copyists frequently added post-script charms and prayers to manuscripts of Jordanus Ruffus and Laurentius Rusius’ treatises, such as a fourteenth-century manuscript with a long procedure against ‘de verme’ invoking the biblical narrative of Job.^64^ Scribes also began incorporating ritual cures into the text of earlier *hippiatric* treatises from the early fourteenth century.^65^ What likely began as annotation and scribal additions then became integrated parts of the treatise through later copying.

Charms were incorporated into the *hippiatric* tradition quickly and became one of the defining features of the texts that followed *De medicina equorum*. A number of these treatises functioned as commentaries on Jordanus and the other earlier writers, and as methods of incorporating Classical learning or experiential knowledge. The remedy collection of Jacopo Doro functioned as an addendum to a horse medicine text written by the famous human surgeon Teodorico Borgognoni.^66^ In addition to remedies for timidness, pain and problems during foaling, Jacopo offered four charms against ‘de verme’ and ‘farcina’.^67^ Boniface of Calabria’s treatise also included several charms and prayers against ‘de verme’.^68^ This was not restricted to texts from Italy: the fourteenth-century French *Cirurgie des Chevaux* included a charm against ‘souros’, debilitating bone spurs that form on the legs, Guillaume de Villiers’ 1456 treatise included several charms and a Sator Square, and Manuel Diez’ *Llibre de Menescalia* (c. 1424–36) contains charms for a number of ailments including *vermis volatilis*.^69^

Horse medicine charms and rituals were of interest to a wide spectrum of readership. Lavishly appointed *hippiatric* manuals were commissioned by aristocratic patrons, including a set of illustrated codices associated with Niccolò III d’Este, the condotierro and marquis of Ferrara. In 1422, Niccolò commissioned a translation of Laurentius Rusius’ *Liber de signis bonitatis et malicie equorum* into Italian.^70^ It is also likely that he was associated with three manuscripts dating to around 1420 that collated several treatises around Boniface of Calabria’s *Libro de la merescalcaria de li cavali*.^71^ At least one text in each of these manuscripts is fully illustrated, with a miniature for each chapter. The illuminations in *M* correspond with the figure style, technique and blond hair of an early fifteenth-century manuscript of the Flower of Battle, which was also commissioned by Niccolò. This codex was part of the library of Giovanni Maria dalla Salla, stablemaster to Alfonso I d’Este (d. 1534) and may well have been passed to them by Alfonso’s grandfather Niccolò.^72^ Several of the treatises in *M* and *BL* including Boniface’s *Libro de la merescalcaria* correlate closely with another early-fifteenth century horse medicine manual, which is even more fully decorated with miniatures depicting a wide range of horses, maladies, practitioners and treatments.^73^ Although the provenance for several of these manuscripts is unclear, their extravagant appointments and the focus of their texts point to a wealthy, chivalric clientele. This group of manuscripts reflects an aristocratic audience who owned *hippiatric* treatises alongside other didactic literature appropriate to the military class. It also mirrors the trend for producing elegantly decorated medical books as high-status items for elite laypersons.^74^ Several of the manuscripts cited above include ritual remedies for infectious diseases; *V* in particular stands out for a prayer against farcy illustrated with a miniature of Job who is stricken with weeping sores and blessing a similarly afflicted horse.^75^

Rituals against infectious diseases in horses were also common in what might be thought of as popular texts: remedy books and collections of treatises prepared by professional horse carers and other people with a vested interest in healing animals. As with higher-status manuscripts, these were often agglomerations of disparate remedies and horse-lore formed around a central treatise. Later users would add remedies, charms and other materials to the text in marginal notes and postscript collections. The importance of disease rituals to audiences who cared for horses is particularly visible in this context as magical remedies against infectious diseases make up a significant proportion of these textual additions. These collections sometimes appear to have been written by several generations of end users, such as an early fifteenth-century English manuscript formed around the *Boke of Marchalsi* with several discrete sections of supplementary remedies added in at least four additional late-medieval hands.^76^ Similar remedy collections are found in a number of vernacular traditions including French, Anglo-Norman, Sicilian and Italian.^77^ As the next section will demonstrate, many of the same formulae and incantations are found in texts from across western and southern Europe, suggesting a robust network of distribution and translation. The broad prevalence of ritual remedies against diseases like farcy in veterinary texts demonstrates that contagious disease was one of the core concerns for medieval horse carers and that the use of veterinary magic was common across the societal spectrum—that charms and rituals were not relegated to low-status horse owners and veterinary practitioners.

## Curing the Worm

The rest of this article will identify some of the distinctive features of rituals dedicated to combatting infectious diseases in horses and explain why these diseases so commonly attracted magical cures. The first task is made more difficult by the quite substantial corpus of disease rituals in *hippiatric* treatises and other veterinary sources and their wide chronological and geographical distribution. Nevertheless, we can identify enough shared features, common formulations, and a canon of familiar *historiolae* and imagery to begin classifying these procedures as a distinctive, discrete healing methodology. In this sense, disease rituals could be thought equivalent to the birthing girdles and apotropaic gemstones that aided childbirth, or the profusion of magical cures for bleeding such as the *Caro*, Longinus and Veronica charms.^78^ Isolating this particular ritual type and considering why it was such a common response to contagious horse diseases helps us to understand the wider mechanisms of veterinary and medical choice and why certain afflictions were more likely to attract magical remedies. By placing these charms and rituals—for the first time—into a wider context of veterinary and medical care we can counter the argument that these were superstitious practices of last resort and emphasise the fundamental place of magic and ritual healing within medieval horse medicine. We can see how magical procedures that harnessed the healing potential of Christ’s resurrection and the occult powers of words and symbols coexisted with curative practices of observation, isolation and dietary control.

Healing rituals were often used as part of a wider strategy for treating disease that included pharmaceutical preparations and surgical interventions. Boniface of Calabria noted that farcy could be treated using diverse methods including medicine and incantations.^79^ A remedy from the collection of Ubertus de Curtenova used crushed poppy seeds alongside three *pater nosters* spoken into the right ear.^80^ The *Boke of Marchalsi* includes a remedy for farcy that involves a long charm performed over several days followed by instructions on cutting pustules and applying a poultice.^81^ In addition to its magical action, this procedure forced the charmer to isolate and observe the horse, keeping watch on its condition and the development of its symptoms. Some charms involved performing therapeutic blood-letting as part of the procedure. Charmers would also restrict the horse’s diet, such as an Anglo-Norman remedy that instructed its reader not to feed or provender the horse or let them cross running water for three days.^82^

The simplest rituals consisted of a prayer or blessing directed to God, the Holy Trinity or a suitable holy figure such as Job or Hippolytus perhaps accompanied by the *ave maria* or *pater noster*. The earliest veterinary charms appended to *hippiatric* texts, such as the collection of Jacopo Doro attached to a copy of Jordanus Ruffus’ *de medicina equorum* from around 1275, often added a few additional flourishes to this paradigm. Doro’s treatise contains four rituals against worms or farcy; a mass for St. Hippolytus, two adjurations via Job and the golden oriole ligature mentioned above.^83^ Later remedies were often much more elaborate with additional tropes, performative elements and *historiolae*.^84^ Some particularly complex fourteenth- and fifteenth-century examples involved multiple stages and holy narratives as well as blessed objects and other healing rituals.^85^ Most of these elements were embedded with recognisable curative resonances that reflected the perceived nature of the disease or related it to scriptural passages, holy figures and narratives associated with healing. Other elements were connected with familiar pharmaceutical or medical methodologies or reflected local healing cultures—particularly processes of miracle-seeking.^86^

The most common feature of charms against farcy was a reflection of the perceived aetiology of the illnesses themselves, which due to their fissure-like pustules were characterised as worms burrowing under the surface and destroying the body. Charmers would often exorcise these worms directly; ‘I adjure you worms, by the Father and the Son and the Holy Spirit’.^87^ To heal the horse of its disease the worms had to be destroyed and cast out of the body to ‘die on the ground’.^88^ Charms explicitly reflected the anxious prognosis that if the worms were not killed and cast out of the body they would spread into the horse’s head or down into its legs and lower extremities.^89^ To prevent this, charmers treated the worms as a physical enemy that must be forced to stand their ground and be destroyed: ‘you will remain where you are [in the horse’s body] and be held and seized and you will die by the power of our Lord’.^90^ Disease charms often assaulted the worm with the incantation ‘the worm is dead, vanquished by the lion of Judah, the line of David, the root of Jesse, the shining star of the morning’^91^. This channelled the redemptive power of Christ through the fulfilment of the prophecy of Isaiah: ‘and there shall come forth a rod out of the root of Jesse’.^92^ Charmers also used a variation of this formula which references Christ opening the Book of Life in Revelation 5: ‘Behold! The lion of the tribe of Judah…has prevailed’.^93^ A similar formulation was used in pseudo-Arnaldus’ *De sigillis* as an inscription on the ‘Lion Seal’, which the historical Arnaldus of Villanova (c. 1240–1311) used to treat Pope Boniface VIII (c. 1230–1303) for kidney stones.^94^ These formulae functioned as *historiolae*, connecting Christ’s power over death with the charmer’s ability—through Christ—to defeat disease.

The characterisation of these diseases as a worm or serpent lent itself to association with the story of Job who was assaulted by worms and other calamities to test his faith in God.^95^ Charms against infectious diseases often reflected on Job’s suffering and applied it to the horse’s condition. Some rituals offered prayers to Job as a reflection of his unwavering faith and forbearance in the face of suffering, whilst others adapted his narrative to apply it more directly to the particular ailment they were treating. As Job was delivered from the torment of worms so too should the charmer’s equine patient be delivered from ‘talpa or farcy and any other evil’.^96^ A fourteenth-century French treatise identified the worms that chewed on Job’s face as the ‘talpin’ worms, which were the cause of the disease that this charm alleviated.^97^ English charms often involved a counting formula in which ‘St Job had nine worms that grieved him much’ and each time he prayed one worm was removed until none remain.^98^ The same charm recounts that Job, even in his torments, asked God to spare his animals from their maladies. The specific comparisons that these charms drew acted as a re-scripting of Job’s narrative, an ‘active analogising’ which is a familiar part of the functionality of charm *historiolae*, in which ‘myth and present actuality are brought into the same space’.^99^ Charmers were active storytellers who developed a structured narrative from Biblical precedents and recognisable formulae.^100^ These narratives did not need to hold up to theological scrutiny as long as they followed certain generic expectations; it was less important whether Job was afflicted with ‘talpin’ worms, and more important that the two situations were paradigmatically similar.

Both physicians and horse healers used charms that referenced familiar biblical figures with a particular relevance to the patient or infirm animal. When treating sick and injured horses, charmers would often reference the Virgin Mary, the archangel Michael who protected Daniel in the Lion’s den, Martha of Bethany—the sister of Lazarus whom Jesus raised from the dead, and Nicodemus who removed the nails from Jesus’ hands and feet.^101^ As with Job, these were usually associated with narratives that suited the needs of the charmer in providing precedent or analogy for the healing action of the charm. Other saints were selected because of a more specific narrative analogy between the holy figure and either disease or the care of horses. St. Nicasius (d. 407), who features in several Middle English charms against farcy, was also associated with the cure of smallpox—another disfiguring disease involving pustules in the skin.^102^ St. Maturus was according to common tradition beheaded in 177 and for the 9 days after he died his body was miraculously unsullied by worms.^103^ St. Hippolytus’ legend adapted over time from one of martyrdom via wild beasts to a narrative that was more closely aligned with horse medicine and stable mastery. He was a third-century theologian at the Church in Rome who was exiled in 235 and likely died in the mines of Sardinia.^104^ However, the legend that was transmitted by Prudentius had him dragged to death by wild horses.^105^ By the fifteenth century, Hippolytus was remembered by horse-doctors as a ‘great stablemaster’ who had knowledge and mastery over horses, granted to him by God whilst he was living amongst them exiled in the wilderness. When King Herod tried to use wild horses to quarter Hippolytus nothing could compel them to do the saint harm, not even the beating of drums and the torment of spears.^106^ As the horses stood firm, Hippolytus prayed to God that their wounds and bleeding should do them no harm but should in fact be therapeutic to them—in the manner of medicinal blood-letting. The progeny of these horses, who by the fifteenth century were thought to reside in Hungary, had learnt to bleed themselves, whenever they had need, which meant that they never suffered from farcy or founder. Hippolytus was often featured in prayers and charms that combatted farcy, founder and other acute illnesses.^107^

Charmers also commonly invoked saint Eligius (d. 660) whose legend was adapted to create an association with horse-care and animal healing long after his death. Eligius was born into an influential Gallo-Roman family and trained as a goldsmith before being made the bishop of Noyon and chief counsellor to Dagobert I, the Merovingian king of France. By the late Middle Ages, he functioned as the patron saint of marshals and horse doctors, who dedicated their guilds to him, illustrated their statutes with his legend and commissioned lavish public art to celebrate him.^108^ His association with horse-care stems from a popular narrative, which arose sometime in the late thirteenth century in which the saint was brought an uncontrollable (or possibly possessed) horse that nobody else was able to shoe. Eligius cut off the horse’s leg around the hock, shod the severed hoof and miraculously reattached the limb. It is unclear when this episode that connected Eligius with horse-care developed as it was not included in his Vita written by Dado of Rouen (d. 684) or miracles.^109^

Eligius’ narrative is most commonly found in visual sources: wall paintings and chancel screens, carvings, tapestries and manuscript illuminations.^110^ The scene was used to illustrate several horse medicine treatises, notably two extensively illuminated copies of Boniface of Calabria’s treatise on horse-care dating from around 1417.^111^ It was also referenced in a Middle English horse medicine collection from the mid-fifteenth century as part of a charm ‘to make a horse stand still’. The treatise presented the legend as part of the *historiola* of the charm; saying that this was the ‘same charme that Seynt Loye hadde when þat he first hors shodde’.^112^ Although this charm is not a response to infectious disease, it demonstrates how veterinary practitioners sought to harness the power of the saints and their legends in their use of charms and prayers. In adapting Eligius’ narrative, the text takes the foundational myth of the horse healer and modulates the miraculous function—in which Eligius’ power comes from his close connection with God—so that the saint’s art and power could be wielded by any marshal and could be explained and disseminated verbally. This meant that as well as developing a professional identity through Eligius, marshals were able to claim and utilise his healing power as their own through practices that were common to their trade—charms and rituals.

Disease rituals also utilised the power of faith and grace through holy water, blessed items and the use of sacrificial tokens. One such remedy is found in a horse medicine collection donated to St Augustine’s Abbey in Canterbury around 1325, which was prescribed for snakebites. It instructed the charmer to take holy water and pass it through the foetal membrane of a cat three times whilst reciting an incantation involving the Trinity and three of the ‘divine names’ of God: ‘Sabaoth, Emanuel, Paraclitus’.^113^ Healers would also feed their horses water or food that had been blessed or otherwise fortified with power. One fifteenth-century Italian charm involved inscribing bread with the words ‘semuz, memuz, cefat, mifat’ before feeding it to the horse, bleeding it from its neck, and covering its coat with mud.^114^ Another instructed the user to recite an incantation referencing several of the names of Jesus (‘the source of strength, the lion, the eagle, the servant’) and then feed the horse thrice-boiled holy water.^115^ A roughly contemporary Middle English remedy for founder involved blessing a handful of oats and sprinkling them with both holy water and holy wax.^116^ In each of these examples, the charmers were utilising the grace or ‘qualitative holiness’ of these substances to heal their horses. Matthew Milner argued that remedies such as these were grounded in a vernacular, natural–philosophical understanding of grace as a transferable Aristotelian quality. This means that the use of saints’ names, blessed food, and holy water in healing remedies was akin to but separate from the dynamics of miraculous healing.^117^

Ritual healers also used candles and other sacrificial tokens in a manner redolent of miracle-seeking practices. A fifteenth-century remedy for farcy attributed to ‘Hew Saracyn of Spayne’ directed the charmer to light three candles in honour of St. Fermin, recite a narrative charm and then make an offering of the candles in Fermin’s name. Another remedy in the same manuscript instructed the user to take a penny belonging to the horse’s owner and dedicate it to St. Charity before performing an elaborate charm and giving the penny to a leper.^118^ A third remedy against worms from a later treatise in that manuscript ends with the charmer bending a penny ‘on the horse’s head in the worship of Saint Loy [Eligius]’.^119^ Candles and other wax objects were common offerings given as part of miracle-seeking rituals, which in the later Middle Ages included a significant number of animal-healing miracles, whereas pennies—and in particular the ritual of penny-bending—seem to have been peculiar to England.^120^ To the horse carers, there was a clear relationship between charming and miracle-seeking praxis. The third remedy above begins with an invocation that suggests that charmers in some sense sought to co-opt the power of the saint-thaumaturge through the familiar rituals of miracle-seeking by direct supplication to God: ‘Lord that sent power into stone and word and grass send power into my words.’^121^

The last common curative for infectious horse diseases that will be dealt with here is one that seems to be unique to this corpus: the affixing or implantation of objects under the skin of the horse. This method is apparent from at least as early as the late-thirteenth century and is found in remedy collections, *hippiatric* treatises, and even in an addendum to a fifteenth-century copy of Frederick II’s famous hunting manual.^122^ There are at least two subgroups of these remedies: one in English and the other in Latin, the latter is found in manuscripts from England, France and Italy—including in texts whose principal language is Italian. This remedy only seems to have been used when treating some form of *vermis* or farcy. One of the earliest iterations is found in a copy of the anonymous *practica equorum* from around 1280:

Against worms in any part of a horse: Cut the skin in the forehead and place two metal sheets there in this fashion one made of lead and the other of tin and you should do this whilst the sun is setting.^123^

Other versions of this remedy involved inscribing words and figures on the metal plates before implanting them into the horse. For instance, an iteration from a fifteenth-century copy of Laurentius Rusius’ *hippiatric* treatise instructed the user to combat ‘the worm that is called farcy’ by inscribing a single lead coin with a figure on each side; a vaulted structure like a church on one face and the words ‘not sat’ on the other. The healer then cut open the horse’s forehead with a goat’s horn and placed the coin under the skin.^124^ A similar procedure against farcy from a fifteenth-century Middle English horse remedy collection has the healer inscribe the side facing the horse with ‘jhezuz nazarenus et iudeus crucis misereri mei’, and the side facing outwards with ‘super aspidem et basiliscum ambulabis et conculcabis’.^125^ The second phrase, ‘you shall walk amongst the asps and the basilisks and you will crush them under foot’ is taken from Psalm 90. Like several of the charms discussed earlier, this procedure invoked the sympathetic relationship between the worm, which characterised these diseases and the snakes and basilisks over which Jesus triumphed. Implanted objects were also inscribed with nonsensical words, which were likely meant to resemble Hebrew.^126^ An implanted charm from a Middle English collection instructs the healer that:

These words should be writ in lead and then set in the horse’s head.

+ Zaron + Zeronen + Zeronem + Betonent + Astra + Lubia +^127^

These words contain familiar stems and morphemes that we see in other charms and in particular ligatures: such as in one against farcy ‘+⋆⋆alamat+⋆zarabatan ⋆Betonroy + ⋆ orthodoy’.^128^ This group of implanted charms were potentially related to talismans or the wearing of relics, but seem most similar to laminas, which were used in human medicine to treat wounds, infertility and other ailments.^129^ Laminas were made of thin metal or sometimes parchment and were closely related to charms and ligatures in their use of language, incantation and ritual. The fundamental distinction between laminas and horse-charm plaques is that laminas were usually hung around the neck or over the afflicted area of the body, and not implanted into the patient. It is unwise to presume that this reflects a greater accepted level of suffering in animal medicine—human surgical remedies after all could involve a significant amount of pain and trauma—but it does distinguish these procedures from related healing traditions.^130^

In addition to metal plaques, healers were also instructed to form objects from herbal and animal products to implant into horses. The *Boke of Marchalsi* instructed the healer to form a cross from the roots of red dock and nettles and then tie these to crosses made from lead and leather. This object was then blessed with a charm for Jesus and St. Job and stitched into a wound which the healer had cut into the horse’s rump. This process created a strong sympathetic link between the horse and Jesus, but unlike other charms which compared farcy with the torments inflicted on Christ or Job, this procedure required that the horse be metaphorically crucified by its handler in order to create the suitable analogical relationship. The horse was wounded by the healer just as Christ was wounded by Longinus at the crucifixion and it was joined with a cross ‘just as you joined your precious body to the holy cross for the salvation of your people’.^131^ The combination of three crosses reflects both the Trinity and the three crosses at Calvary. The use of red dock is reminiscent of an earlier remedy against farcy from the thirteenth-century Latin *Practica equorum*, which instructed the healer to take red dock root and place it under the skin of the horse’s forehead; if by the same time the next day it had gone then the horse will be healed.^132^ The disease name (‘farcin’) and the curative (‘radice rubee parelle’) in this are both in French, and this section of the treatise contains several words for healing that have been imperfectly adapted into Latin from French (‘garrire’ and ‘sainatet’). These examples of the implanted object charm are only found in texts from England, so perhaps reflect a localised tradition which was transmitted from Anglo-Norman Latin into Middle English.

The different iterations of this cure are similar enough in their targeted ailments and methodologies to justify discussing it as a cohesive tradition, but the variations between texts remind us of the instability of the categorisations that we might apply to medieval remedies. When the implantation remedy incorporates a verbal incantation or inscription, we can comfortably think of it as a charm or talisman. When it involves both lead and tin, we might infer that it was thought to work through the occult properties of different substances. The sympathetic relationship with the suffering of Christ is reminiscent of the use of the *arma Christi—*veneration of the wounds of Christ—in birthing girdles and other aids to parturient women.^133^ The inherent similarities between implantation remedies and laminas could sensibly lead us to see a relationship between the two traditions or to investigate amulets, astral magic, and other magical figures and images. Some of these connections are certainly valid, but just as scholars of magic have cautioned against shepherding disparate curatives into slippery categories such as ‘superstitious’ or ‘magical’, so too should we be cautious not to ignore the distinctiveness of individual remedies.^134^

## Conclusions

The speed with which rituals against farcy were incorporated into the textual culture of western horse medicine in the late Middle Ages speaks to a real need for this material. The diversity and complexity of these remedies speaks to the threat presented to horses by contagious disease, particularly when read in concert with evidence for frequent occurrences of disease at royal stables. The relationships that these rituals constructed between farcy and biblical narratives of suffering, redemption, and resurrection reflected just how dangerous and destructive this disease could be. Animal illnesses have often not been given sufficient attention both for their socioeconomic impact and as motivators for healing cultures in the later Middle Ages. Whilst studies of widespread epizootics have demonstrated the catastrophic loss of animal life and concomitant economic depression that these outbreaks could cause, enzootic diseases like farcy have largely been ignored.^135^ Farcy was particularly hazardous because of its potential to cause serious illness and death, its propensity for spreading amongst and between groups of horses, and its ability to be transmitted to humans.

All of this meant that horse carers needed serious medical responses and therefore developed a distinctive set of ritual practices that were informed by natural philosophy, biblical semiotics, and miracle-seeking practices. Because farcy was one of a set of diseases with the worm as central aetiology this allowed for a cohesive barrage of similarly-functioning charms and rituals to be applied to all of them. These diseases were constructed in a fashion that lent themselves to healing by performative ritual; the adversarial characterisation of the disease as worm and the horse as forbearing patient was a powerful and often-used narrative configuration that allowed horse carers to invoke the suffering of Job and Christ. Healers often placed the horse within the ideals of grace and redemption: when they invoked Nicodemus removing the nails from Jesus’ hands and feet, they painted the horse firmly in the role of Christ as holy sufferer.^136^ Far from being excluded from divine healing magic because of their status as beasts, horses were comfortably incorporated into Christian prayer.

It is revealing that charms and other rituals were most commonly brought to bear against enzootic diseases like farcy, which would take perhaps a few animals in a population each year, rather than the large epizootic outbreaks of quinsy or summer fevers which struck less frequently but had a much greater mortality. For marshals, horse dealers and anybody else who cared for horses, enzootic disease would have been a familiar problem. Using charms against farcy, therefore, was within the realm of everyday rather than peculiar practices, a response by horse carers to a regular and persistent frustration. These remedies were equally persistent: they were copied widely, adapted and found in a substantial proportion of horse medicine texts. Charms and rituals against farcy and other contagious horse diseases would have been recited and performed regularly. The variety of ritual treatments against farcy as well as the persistent use of particular themes and mechanisms speaks to the tenacity of a broad healing tradition that should be read alongside childbirth rituals and remedies for bleeding as one of the key manifestations of late-medieval healing magic.

## Acknowledgements

The author would like to thank the reviewers for their direction and support, Iona McCleery for comments on an early draft, and Kathleen Walker-Meikle for judicious advice regarding venomous snakes.

## Manuscripts Cited

**Barcelona**

Biblioteca Catalunya. MS 1661

**Bologna**

Biblioteca dell’Archiginnasio, MS A1525

**Cambridge**

Corpus Christi College, MS 297 fols. 203–205^v^

Corpus Christi College, MS 301, fol. 81

University Library, MS Additional 3120, fols. 113–117

University Library, MS Dd. IV. 44

**Cesena**

Biblioteca Malatestiana, MS S.XXVI.2

**Florence**

Biblioteca Medicea Laurenziana, MS Plut.77.25

Biblioteca Nazionale Centrale, MS Fonde Nazionale II.02.067

Biblioteca Riccardiana, MS 2934, fols 40–57v

**Lincoln**

Cathedral Library, MS 211, fols. 27–27^v^

**London**

British Library, MS Additional 15097 (*B*)

British Library, MS Additional 22126

British Library, MS Additional 22824

British Library MS Additional 33996, fols. 211–212v

British Library MS Additional 35179, fols. 20v–30v

British Library, MS Harley 3535, fols 2–36

British Library, MS Harley 6398, fols. 28v–29v

British Library, MS Sloane 962, fols. 133v–137

British Library, MS Sloane 3285, fols 88v–92v

Wellcome Library, MS 546, fols. 78v–89

Wellcome Library, MS 700

Wellcome Library MS. 5650, fols 1–28v

**Luxembourg**

Bibliothèque nationale, MS 109, fol. 300^r^

**Modena**

Biblioteca Estense MS Ital. 464 (E)

Biblioteca Estense MS Lat. 111.

**Nantes**

Musée Thomas-Dobree MS 19 f. 220v

**Naples**

Bib. Girolamini. MS Cf.2.7

**New Haven**

Yale University Beinecke Library, MS 488

Yale University Beinecke Library, MS 788, fols. 28v–38

Yale University Beinecke Library, MS 917

Yale University Beinecke Library, MS 1024, fols. 53–65

**New York**

Morgan Library, MS M735 (*M*)

Vatican City

Biblioteca Apostolica Vaticani, MS. Reg. Lat 1010 (*R*)

Biblioteca Apostolica Vaticani, MS Urb. Lat. 1014

Biblioteca Apostolica Vaticani, MS Vat. Lat. 4475

Biblioteca Apostolica Vaticani, MS Vat. Lat. 7228 (*V*)

**Venice**

Biblioteca Marciana, MS Ital. III 27

Biblioteca Marciana, MS Lat. VII 24

Biblioteca Marciana, MS Lat. VII 25

## Footnotes

1 Peter Murray Jones and Lea T. Olsan, ‘Medicine and Magic’, in Sophie Page and Catherine Rider, eds, *Routledge History of Medieval Magic* (London: Routledge, 2019), 308.

2 László Sándor Chardonnens and Rosanne Hebing, ‘Two Charms in a Late Medieval English Manuscript at Nijmegen University Library’, *The Review of English Studies*, 2011, 62, 183; Lea T. Olsan, ‘Charms and Prayers in Medieval Medical Theory and Practice’, *Social History of Medicine*, 2003, 16.

3 Catherine Rider, ‘Medical Magic and the Church in Thirteenth-Century England’, *Social History of Medicine*, 2011, 24, 103–04.

4 Matthew Milner, ‘The Physics of Holy Oats: Vernacular Knowledge, Qualities, and Remedy in Fifteenth-Century England’, *Journal of Medieval and Early Modern Studies*, 2013, 43, 219–21; Peregrine Horden, ‘What’s Wrong with Early Medieval Medicine?’, *Social History of Medicine*, 2011, 24, 8.

5 Yves Lignereux, ‘Les Soins Vétérinaires aux Cheveaux au Moyen Âge’, in Mireille Mousnier, ed, *Les animaux malades en Europe occidentale (VIe-XIXe siècle): actes des XXVes Journées internationales d’histoire de l’abbaye de Flaran, 12, 13, 14 Septembre 2003* (Toulouse: Presses Universitaires du Mirail, 2005), 50.

6 Bernard Ribemont, ‘Science et magie: La therapie magique dans l’hippiatrie medievale', in Danielle Buschinger and Wolfgang Spiewok, eds, *Zauberer und Hexen in der Kultur des Mittelalters* (Griefswald: Reineke-Verlag, 1994), 189–90.

7 Tony Hunt referred to several remedies added to a Middle French horse medicine text as ‘[SPURIA]’ in his edition of Wellcome 546, *Old French Medical Texts* (Paris: Classiques Garnier, 2011), 269.

8 Antonio Montinaro, ‘Per La Tradizione Del De Medicina Equorum de Giordano Ruffo (Con Un Elenco Dei Testimoni Manoscritti)’, *Medioevo Lettarario d’Italia*, 2010, 7, 37 ‘…alla crescente utilizzazione del cavallo da parte di ceti meno abbienti non per usi militari ma in stretto rapporto con le attività quotidiane.’

9 ‘une science excluant toute “tache”’, Ribemont, ‘Science et magie’, 190; Roberto Benedetti, ‘La Mascalcia di Giordano Ruffo di Calabria secondo le prime redazioni volgari: il caso del frammento 159 dell'Archivio di stato di Udine', in Rita Librandi and Rosa Piro, eds, *Lo Scaffale della Biblioteca Scientifica in Volgare (Secoli XIII-XVI): Atti del Convegno (Matera, 14–15 Ottobre 2004)* (Florence: SISMEL, 2006), 297; Gianvito Resta, ‘Scongiuri in Siciliano in Antichi Trattati Di Masclacia’, in *Aspetti E Prospettive Della Ricerca Demologica in Italia* (Palermo: Manfredi, 1973), 393–94.

10 Milner, ‘The Physics of Holy Oats’, 237.

11 These were distinct from pathological discussions of parasitic worms in horses, which had their own analyses and remedies.

12 Klaus-Dietrich Fischer, ‘“A Horse! A Horse! My Kingdom for a Horse!” Versions of Greek Horse Medicine in Medieval Italy’, *Medizinhistorisches Journal*, 1999, 34, 123–38; Domizia Trolli, *Studi su antichi trattati di veterinaria* (Parma: Univ. degli Studi, 1990). Yvonne Poulle-Drieux’s overview of the principal texts *Médecine humaine et vétérinaire à la fin du Moyen Age : trois études* (Paris: Librairie Droz, 1966); can be supplemented with Lignereux, ‘Les Soins Vétérinaires aux Cheveaux au Moyen Âge’, 41–56.

13 Cécile Auliard, ‘Les Maréchaux à l’époque Médiévale: Forgerons Ou Vétérinaires?’, *Médiévales: Langue, Textes, Histoire*, 1997, 33.

14 John Langdon ‘Horse Hauling: A Revolution in Vehicle Transport in Twelfth and Thirteenth-Century England?’ *Past and Present*, 1984, 103.

15 Fabienne Meiers, ‘Equestrian Cities: The Use of Riding Horses and Characteristics of Horse Husbandry in Late Medieval Urban Agglomerations’, in Anastasija Ropa and Timothy Dawson, eds, *The Horse in Premodern European Culture* (Boston: De Gruyter, 2019); Andrew Ayton, *Knights and Warhorses: Military Service and the English Aristocracy Under Edward III* (Woodbridge: Boydell Press, 1994).

16 Susan Crane, ‘Chivalry and the Pre/Postmodern’, *Postmedieval: A Journal of Medieval Cultural Studies*, 2011, 2.

17 Andrew G. Miller, ‘“Tails” of Masculinity: Knights, Clerics, and the Mutilation of Horses in Medieval England’, *Speculum*, 2013, 88, 968–69; Francisco LaRubia-Prado, ‘Gift-Giving Diplomacy: The Role of the Horse in the Cantar de mio Cid’, *La corónica: A Journal of Medieval Hispanic Languages, Literatures, and Cultures*, 2008, 37; Charles Gladitz, *Horse Breeding in the Medieval World* (Dublin: Four Courts Press, 1997).

18 Auliard, ‘Les Maréchaux à l’époque Médiévale’; Giuseppe Simonetti, *L’Arte Dei Fabbri in Pisa: Statuto Del Secolo XIV* (Rocca S. Casciano: Prem. Stab. Tip. Cappelli, 1894).

19 Farcy was known in Medieval Latin as ‘farcinum’ (for instance in BAV, Reg. Lat. 1010 fol. 17) and may derive from ‘farciminosus’ (i.e. ‘swelling’ or ‘stuffing’) illnesses in Classical veterinary literature, for instance in Ernestus Lommatzsch, ed, *P. Vegeti Renati Digestorum Artis Mulomedicinae Libri*, (Leipzig: B.G. Teubner, 1905), 19. *Farcinum* was rendered into Anglo-Norman as *farcin* and into Middle English as *farcy/farsi*: see Juhani Norri, *Dictionary of Medical Vocabulary in English, 1375-1550: Body Parts, Sicknesses, Instruments, and Medicinal Preparations. Part I* (New York: Ashgate, 2016), 384.

20 The physician Gilbertus Anglicus (d. 1250) described a human canker of the mouth ‘called farcy’ in his *Compendium medicinae*, (Lyon, 1510), VII. 331. 2. Modern health care workers recognise farcy as transmissible to humans; ‘Transmission’, Glanders, CDC, last accessed 24 March 2021, https://www.cdc.gov/glanders/transmission/index.html

21 Nicole Archambeau, ‘Healing Options during the Plague: Survivor Stories from a Fourteenth-Century Canonization Inquest’, *Bulletin of the History of Medicine*, 2011, 85; Abigail Agresta, ‘From Purification to Protection: Plague Response in Late Medieval Valencia’, *Speculum*, 2020, 95.

22 Abigail Woods, ‘From Coordinated Campaigns to Watertight Compartments: Diseased Sheep and their Investigation in Britain, c. 1880-1920’ in Abigail Woods et al., eds, *Animals and the Shaping of Modern Medicine: One Health and Its Histories* (Basingstoke: Palgrave Macmillan, 2018).

23 For instance, Philip Slavin, ‘Epizootic Landscapes: Sheep Scab and Regional Environment in England in 1279–1280’, *Landscapes*, 2016, 17; ‘The Great Bovine Pestilence and its Economic and Environmental Consequences in England and Wales, 1318–50.' *Economic History Review*, 2012, 65; Timothy Newfield, ‘A Cattle Panzootic in Early Fourteenth-Century Europe', *Agricultural History Review*, 2009, 57.

24 A notable exception being Philip Slavin’s discussion of a liturgical response to an outbreak of cattle murrain in England, 1279-80: ‘Flogging a Dead Cow: Coping with Animal Panzootic on the Eve of the Black Death’, in A. T. Brown, Andy Burn, and Rob Doherty, eds, *Crises in Economic and Social History: A Comparative Perspective* (Woodbridge: Boydell, 2015), 120–25.

25 ‘Epizootic’ refers to an outbreak of disease that is temporarily prevalent within an animal population, the human equivalent is epidemic. ‘Enzootic’ is the animal equivalent to endemic and refers to a disease that is persistently apparent within an animal population but usually only effects a few animals at a time. Enzootic diseases are liable to become epizootic—or in fact epidemic—under particular circumstances.

26 Modern veterinary epidemiology still recognises a subcutaneous (farcy) and pulmonary (glanders) form of this disease, Lise Wilkinson, ‘Glanders: Medicine and Veterinary Medicine in Common Pursuit of a Contagious Disease’, *Medical History*, 1981, 25, 364–65.

27 Jordanus Ruffus, *Jordani Ruffi Calabriensis Hippiatrica*, Hieronymus Molin, ed (Padua: Seminarii Patavini, 1818), 27–28.

28 Martina Hurler, *Magister Maurus: Transkription, Übersetzung und veterinärmedizinisch-historische Bedeutung des Manuskriptes aus dem Codex Harleian 3772 der London, British Library* (Königshausen & Neumann, 2007), 119.

29
*Ruffus*, 23–24.

30 Laurentius Rusius, *La mascalcia di Lorenzo Rusio volgarizzamento del secolo XIV*, Pietro Delprato and Luigi Barbieri, eds (Bologna: Presso G. Romagnoli, 1867), 356.

31 See for instance Luis García-Ballester, ‘On the Origin of the “Six Non-Natural Things” in Galen’, in Jon Arrizabalaga et al., eds, *Galen and Galenism: Theory and Medical Practice from Antiquity to the European Renaissance* (Aldershot: Ashgate, 2002)

32 Hurler, ‘Magister Maurus’, 119.

33
*Ruffus*, 23; Hermann Stadler, *Albertus Magnus De Animalibus Libri XXVI Nach Der Cölner Urschrift* (Münster: der Aschendorffschen Verlagsbuchhandlung, 1920), 1389–90; *Rusius*, 356.

34
*Albertus Magnus*, 1390; *Rusius*, 356.

35 London, British Library MS Additional 35179 fols. 21, 25.

36 Cambridge, University Library, MS. Additional 3120, fol. 114v; *Albertus Magnus*, 1386–87; London, British Library, MS Additional 35179, fol. 25.

37 London, British Library, MS Additional 35179, fol. 21.

38 Otherwise known as *morve* or *farsin* and typified like *vermis volatilis* by a discharge of mucous. Yvonne Poulle-Drieux, ‘Pratique de l’hippiatrie à La Fin Du Moyen Age’, in Danielle Jacquart, ed, *Comprendre et Maîtriser La Nature Au Moyen Age: Mélanges d’histoire Des Sciences Offerts à Guy Beaujouan* (Paris: Librairie Champion, 1994), 332.

39 ‘avenam attactu prius reliquiarum S. Magni et sacra precatione lustratam afferunt’, *Acta Sanctorum Septembris Tomus Secundus* (Antwerp: Bernardum Albertum Vander Plassche, 1758) 760.

40 Vatican City, Biblioteca Apostolica Vaticani, MS Vat. Lat. 7228 (*V*), fol. 42.

41
*Ruffus*, 28, 115.

42 Jeffrey Jerome Cohen, *Medieval Identity Machines* (Minneapolis: University of Minnesota Press, 2003), 35–77; For a parallel analysis of the social impact of disfigurement on humans, see Patricia Skinner, ‘“Better Off Dead Than Disfigured”? The Challenges of Facial Injury in the Pre-Modern Past’, *Transactions of the Royal Historical Society*, 2016, 26.

43 ‘Henrico Marescallo pro dicto dextrario nigro coquendo et marecalciando pro farso per vices xiij d. ‘pro stipendio suo’. London, National Archives, E101/97/3, m. 11.

44 London, National Archives, E101/100/35, m. 1 and E101/101/8, m. 2.

45 Etienne Picard, *L’écurie de Philippe le Hardi, duc de Bourgogne: d’après des documents inédits* (Paris: Chez H. Champion, 1906), 93.

46 Paris, Archives Nationales, KK. 53, fol. 68.

47 Rome, Biblioteca Apostolica Vaticani, MS. Reg. Lat 1010, fol. 102 (henceforth, *R*); Kelly-Anne Gilbertson, “A Fifteenth Century Treatise on Horses: A Critical Edition from a Manuscript in a Private Collection” (unpublished Manuscript, University of Johannesburg, 2014), 40, 68.

48 Olsan, ‘Charms and Prayers’, n. 105.

49 Jones and Olsan, ‘Medicine and Magic’, 299–300.

50
*R*, fol. 26; Venice, Biblioteca Marciana, MS Lat. VII 24, fol. 69.

51 Peregrine Horden has argued that medicines derived from animals tend to be overlooked in favour of herbal medicine, because they are less palatable to a modern audience who deem biomedicine—which is largely derived from flora—as ‘real medicine’, ‘What’s Wrong with Early Medieval Medicine?’, 8–9.

52 Lorraine Daston and Katharine Park, *Wonders and the Order of Nature, 1150-1750* (Cambridge, MA: MIT Press, 1998), 127.

53 Venice, Biblioteca Marciana, MS Lat. VII 25, fol. 31^v^.

54 Thomas of Chobham, *Summa confessorum*, F. Broomfield, ed (Louvain: Editions Nauwelaerts, 1968), 477.

55 Horden, ‘What’s Wrong with Early Medieval Medicine?’, 16.

56 Antonio Montinaro, *La tradizione del De medicina equorum di Giordano Ruffo: con un censimento dei testimoni manoscritti a stampa* (Milano: Ledizioni, 2015), 26–28.

57 Venice, Biblioteca Marciana, MS Lat. VII 25, fols. 31-34^v^; *R*, fol. 61–160^v^; Vatican City, Biblioteca Apostolica Vaticani, MS Vat. Lat. 7228.

58 Olsan, ‘Charms and Prayers’, 343–66.

59 London, British Library, MS Additional 15097 (Henceforth: *B*); Vatican City, Biblioteca Apostolica Vaticani, MS Vat. Lat. 7228; Florence, BNCF, Magl. XV 26; New York, Morgan Library, MS M735; Naples, Bib. Girolamini. MS Cf.2.7; Bologna, Biblioteca dell’Archiginnasio, MS A1525.

60 See, Trolli.

61 In addtion to charms and amulets, horse medicine treatises were quick to incorporate divination and astrological instructions including the *equum signorum* or zodiac horse; Josefina Planas, ‘The Zodiac and the Stars in a Treatise on Veterinary Medicine of the Crown of Aragon’, *Manuscripta*, 2012, 56.

62 Venice, Biblioteca Marciana, MS Lat. VII 24, fol. 69.

63 London, Wellcome Library, MS 700, fol. 50^v^.

64 Florence, Biblioteca Medicea Laurenziana, MS Plut.77.25 fols. 50-50^v^.

65 London, British Library, MS Additional 22126; Vatican City, Biblioteca Apostolica Vaticani, MS Urb. Lat. 1014; Modena Biblioteca Estense MS Lat. 111.

66 Theodoric of Cervia, *The Surgery of Theodoric: Ca. A.D. 1267.*, Eldridge Campbell and James Colton, trans. (New York: Appleton-Century-Crofts, 1960); Teodorico’s *hippiatric* treatise was edited in *Die Pferdeheilkunde des Bischofs Theoderich von Cervia: Abhandlung I-III*, trans. Erich Dolz, Günther Klütz, and Wilhelm Heinemeyer, eds, 3 vols (Berlin: Friedrich-Wilhelm-Universität, 1935–6).

67 Venice, Biblioteca Marciana, MS Lat. VII 25, fols. 31–34^v^.

68
*B*, fols. 40^v^–41.

69 Brigitte Prévot and Bernard Ribémont*, Le cheval en France au Moyen Age: sa place dans le monde médiéval; sa médecine, l’exemple d’un traité vétérinaire du XIVe siècle, la Cirurgie des chevaux* (Caen: Paradigme, 1994), 356; Yvonne Poulle-Drieux, ‘L’hippiatrie Dans l’Occident Latin Du XIIIe Au XVe Siècles’, in Guy Beaujouan, ed, *Médécine Humaine et Vétérinaire à La Fin Du Moyen Age* (Paris: Droz, 1966), 119–21; Barcelona, Biblioteca Catalunya. MS 1661, fols. 30^v^, 59, 67–67^v^, 80^v^.

70 London, British Library, MS Additional 22824.

71 New York, Morgan Library, MS M735 (*M*); Modena, Biblioteca Estense MS Ital. 464 (*E*); London, British Library, MS Additional 15097 (*B*); Trolli, *Studi su antichi trattati di veterinaria*, 69–80.

72 New York, Morgan Library, MS M.383, see also the unpublished manuscript notes for MS M. 735 (Morgan Library, 1949), 2.

73
*V*. As we cannot firmly date any of these manuscripts it is difficult to speculate about their respective chronology.

74 Monica Green, ‘Medical Books’, in Eric Kwakkel and Rodney Thomson, eds, *The European Book in the Twelfth Century*, (Cambridge: Cambridge University Press, 2018), 287–90.

75
*V*, fol. 42^r^.

76 Cambridge, University Library, MS Dd. IV. 44 (*D*).

77 For example, London, Wellcome Library, MS 546, fols. 78^v^–89; Cambridge, Corpus Christi College, MS 297 fols. 203–205^v^; Florence, Biblioteca Riccardiana, MS 2934; New Haven, Yale University Beinecke Library, MS 788, fols. 28^v^–38.

78 Katherine French, ‘The Material Culture of Childbirth in Late Medieval London and Its Suburbs’, *Journal of Women’s History*, 2016, 28, 133–36; Peter Murray Jones and Lea T. Olsan, ‘Performative Rituals for Conception and Childbirth in England, 900-1500’, *Bulletin of the History of Medicine*, 2015, 89; Olsan, ‘Charms and Prayers’, 356–60.

79
*V*, fol. 42.

80
*R*, fol. 147^v^.

81
*D*, fols. 5^v^–7.

82 London, British Library MS Additional 33996, fol. 212–212^v^.

83 Venice, Biblioteca Marciana, MS Lat. VII 25, fol. 31^v^.

84
*Historiolae* are narrative of past events relevant to the patient’s current condition, which therefore allowed the charmer to create an ‘analogical relation between the content of the text, and the…curative function of the formula of which it forms a part’. Daniel James Waller, ‘Echo and the Historiola: Theorizing the Narrative Incantation’, *Archiv Für Religionsgeschichte*, 2015, 16, 269.

85 London, British Library, MS Harley 3535, fol. 36^v^ (hereafter, *H*); New Haven, Yale University Beinecke Library, MS 488 fol. 28^v^; Florence, Biblioteca Medicea Laurenziana, MS Plut. 77. 25, fols. 50–50^v^; Rome, Biblioteca Apostolica Vaticani, MS Vat. Lat. 4475, fols. 25–26^v^.

86 The role of miracle-seeking within medieval healing cultures has been well established by, for instance, Rachel Koopmans, *Wonderful to Relate: Miracle Stories and Miracle Collecting in High Medieval England* (Philadelphia, PA: University of Pennsylvania Press, 2011); Nicole Archambeau, ‘Miracle Mediators as Healing Practitioners: The Knowledge and Practice of Healing with Relics’, *Social History of Medicine*, 2018, 31; Claire Trenery, *Madness, Medicine and Miracle in Twelfth-Century England* (Abingdon: Routledge, 2019). In comparison, veterinary miracles have received quite limited attention, notably by Briony Aitchison, ‘Holy Cow!: The Miraculous Cures of Animals in Late Medieval England’, *European Review of History: Revue Européenne d’histoire*, 2009, 16.

87 ‘Ego te adiuro vermis + per patrem + et filium + et spiritus sancti’, Rome, Biblioteca Apostolica Vaticani, MS Vat. Lat. 4475, fol. 26.

88 ‘nunquam vivificentur’ London, British Library, MS Additional 33996, fol. 212^v^; ‘ki stu vermis mortu in terra vaia et dissinda’ Aldo Fichera, ‘L’edizione Dei Due Trattati Di Mascalcia in Volgare Siciliano Del Codice 2394 Della Biblioteca Riccardiana Di Firenze’ (unpublished Tesi di Dottorato, Università degli Studi di Catania, 2015), 74.

89 ‘ut non amplius facias lesiones ad istum equum nec in capitem equi ascendatis nec in cruram nec in alia membra’ *H*, fol. 36^v^; ‘ibi arrescat nec crescat nec in capud eius assendat nec in pedibus eius dessendat’, Fichera, ‘Due Trattati Di Mascalcia’, 212.

90 ‘in loco in quo te inveni per maneas et arrestras et moriaris per virtutem domini nostri Jhesu Christi’, *H*, fol. 36^v^.

91 This invocation is found in Anglo-Norman in London, Wellcome Library, MS 546, f. 88r as well as several Latin and Sicilian examples: *H*, fol. 36^v^; Florence, Biblioteca Riccardiana, MS 2394 fol. 49^v^; Venice, Biblioteca Marciana, MS Ital. III 27, fols. 16^v^–17.

92 Isaiah 11.1.

93
*V*, fol. 42^v^.

94 Don C. Skemer, *Binding Words: Textual Amulets in the Middle Ages* (University Park, PA.: Pennsylvania State University Press, 2006), 132.

95 ‘My flesh is clothed with worms and clods of dust; my skin is broken, and has become loathsome’, Job 7.5.

96
*B*, fol. 41.

97 London, Wellcome Library, MS 546, fol. 88.

98
*D*, fols. 6v

99 Waller, ‘Echo and the Historiola’, 270.

100 The practitioner was considered a key part of the success or failure of ritual healing procedures, Maaike van der Lugt, ‘The Learned Physician as a Charismatic Healer: Urso of Salerno (Flourished End of Twelfth Century) on Incantations in Medicine, Magic, and Religion’, *Bulletin of the History of Medicine*, 2013, 87.

101
*H*, fol. 36^v^, Lincoln, Cathedral Library, MS 211, fol. 27^v^; W. L. Braekman, *Of Hawks and Horses: Four Late Middle English Prose Treatises* (Brussels: Omirel, 1986), 96; *B*, fol. 40^v^-41, Luxembourg, Bibliothèque nationale, MS 109, fol. 300^r^, New Haven, Yale University Beinecke Library, MS 917, fol. 47^v^.

102
*D*, fol. 27^v^; London, British Library, MS Sloane 3285, fol. 89^v^. For Nicasius and smallpox see London, British Library, MS Cotton Caligula A. XV, fol. 129.

103
*V*, fol. 42v.

104
*On the Apostolic Tradition*, Alistair Stewart-Sykes, ed. (Crestwood, NY: St. Vladimir’s Seminary Press, 2001).

105
*Prudentius*, Henry John Thompson, trans. (Cambridge, MA: Harvard University Press, 1953), II, 305–13.

106 Bengt Odenstedt, *The Boke of Marchalsi a 15th Century Treatise on Horse-Breeding and Veterinary Medicine: Edited from MS. Harley 6398* (Stockholm: Stockholm University, 1973), 21–23.

107 Venice, Bibioteca Marciana, MS. Lat. VII 25, fol. 31^v^; *R*, fol. 146; London, British Library, MS Sloane 3285, f. 90^v^–91.

108 Joyce W. Percy, *York Memorandum Book* (Gateshead: Northumberland Press, 1973), 178; Augustin Thierry, *Recueil des monuments inédits de l’histoire du Tiers-Etat. Première série, Chartes, coutumes, actes municipaux, statuts des villes et communes de France. Région du Nord* (Paris: Imprimeurs de L’institut de France, 1850). II, 467; Lia Brunori Cianti and Luca Cianti, ‘Lo strumentario podologico nella mascalcia medievale. Testi e iconografia’, *Pallas. Revue d’études antiques*, 2016, 101, 147 n. 14; Giovanni Antonio di Banco, Tabernacolo dell'Arte dei Maniscalchi (Florence, 1417–21).

109 Ludovic Notte, ‘La Dévotion À Saint Éloi Dans Les Écuries Princières (XIIIe-XVIe Siècles)’, *Revue Belge de Philologie et D’histoire*, 2003, 81, 1052.

110 Andrew Breeze, ‘Chaucer, St. Loy, and the Celts’, *Reading Medieval Studies*, 1991, 17.

111 See Frederic Madden, Catalogue of Additions to the Manuscripts in the British Museum in the Year MDCCCXLI-MDCCCXLV (London: Printed by order the Trustees, 1850), 86; Bernhard Degenhart and Annegrit Schmitt, *Corpus der italienischen Zeichunungen, 1300-1450* (Berlin: Gebr. Mann Verlag, 1980), II/2, 349, 392–415, no. 716, figs. 646, 649, 652, 655, 658, 661, pls. 195–201. Other horse medicine treatises that use the Eligius iconography include Cesena, Biblioteca Malatestiana, MS S.XXVI.2 and Oxford, Bodleian Library, MS. Auct. 4Q 5.69 which was printed in Venice in 1493.

112
*D*, fol. 25^v^.

113 Cambridge, Corpus Christi College, MS 301, fol. 81. Don Skemer has argued that divine names which were transliterated from Hebrew and Greek, such as the three used here as well as *Tetragrammaton*, *Adonai*, and *Agios*, were the most potent: Skemer, *Binding Words*, 112.

114 New Haven, Yale University Beinecke Library, MS 488, fol. 38.

115 Florence, Biblioteca Nazionale Centrale, MS Fonde Nazionale II.02.067, fol. 186.

116 London, British Library, MS Sloane 3285, fols. 90^v^-91.

117 Milner, ‘The Physics of Holy Oats’, 233.

118
*D*, fol. 5^v^-7; another charm involving the sacrificial offering of a penny can be found in Braekman, *Of Hawks and Horses*, 92.

119
*D*, fol. 34^v^.

120 Robert Bartlett, *Why Can the Dead Do Such Great Things? Saints and Worshippers from the Martyrs to the Reformation* (Princeton: Princeton University Press, 2013), 355–56.

121 ‘Lord that sendist vertu in stone in worde in grasse send vertu in my wordes’.

122 Nantes, Musée Thomas-Dobree MS 19, f. 220v.

123 ‘Ad vermes equi alicubi. Finde cutem in frontem pone duas lammas ad hunc modum ■■ quarum una sit plumbea et alia de ere albo et haec facias in occasu solis’, Lincoln, Cathedral Library, MS 211, fol. 27. This remedy is also found in *R*, fol. 2^v^ and New Haven, Yale University Beinecke Library, MS 1024, fol. 57.

124 London, British Library, MS Additional 22126, fol. 11.

125 Braekman, *Of Hawks and Horses*, 92.

126 Richard Kieckhefer, *Magic in the Middle Ages* (Cambridge: Cambridge University Press, 1990), 28.

127
*D*, fol. 35.

128 London, British Library, MS Additional 22126, fol. 11.

129 Sophie Page, ‘Medieval Magical Figures: Between Image and Text’, in Sophie Page and Catherine Rider, eds, *Routledge History of Medieval Magic* (London: Routledge, 2019), 435.

130 Nancy Siraisi discussed the role of pain in deciding whether to undergo surgery in *Medieval and Early Renaissance Medicine: An Introduction to Knowledge and Practice.* (Chicago: University of Chicago Press, 1990), 153–54.

131 London, Wellcome Library MS. 5650, fols. 14^v^-16; see also *D*, fols. 6–7 and London, British Library, MS Harley 6398 fols. 28^v^–29^v^.

132 Lincoln, Cathedral Library, MS 211, fol. 27^v^.

133 Jones and Olsan, ‘Performative Rituals for Conception and Childbirth’, 426–27.

134 Milner, ‘The Physics of Holy Oats’, 220.

135 Philip Slavin estimates that during the 1275–80 outbreak of sheep scab and the Great Bovine Pestilence of 1319–20 in England, the mortality rates for sheep and cows reached 50 and 62 per cent, respectively, and the painfully slow recovery from these events caused serious economic hardship, ‘Mites and Merchants: The Crisis of English Wool and Textile Trade Revisited, c. 1275–1330’, *Economic History Review*, 1–33 (8, 18) [ONLINE PREPRINT].

136 New Haven, Yale University Beinecke Library, MS 917, fol. 47^v^.

## Notes

Sunny Harrison works on the history of veterinary medicine and animal care in the Late Middle Ages. His first monograph, due in 2022 with Amsterdam University Press’ Premodern Health, Disease, and Disability series, will chart the development of horse medicine in late-medieval western Europe and its relationship with the theories and practices of human medicine. He is currently an Associate Lecturer with the Open University.

